# Supplementary material for: Dysbiosis in the Nasal Mycobiome of Infants Born in the Aftermath of Hurricane Maria
Source: Microorganisms. 2025 Jul 31;13(8):1784. doi: 10.3390/microorganisms13081784 (PMC12388473; doi:10.3390/microorganisms13081784)
Supplement: Supplementary file 1 [file microorganisms-13-01784-s001.zip › microorganisms-3739908-supplementary.pdf]

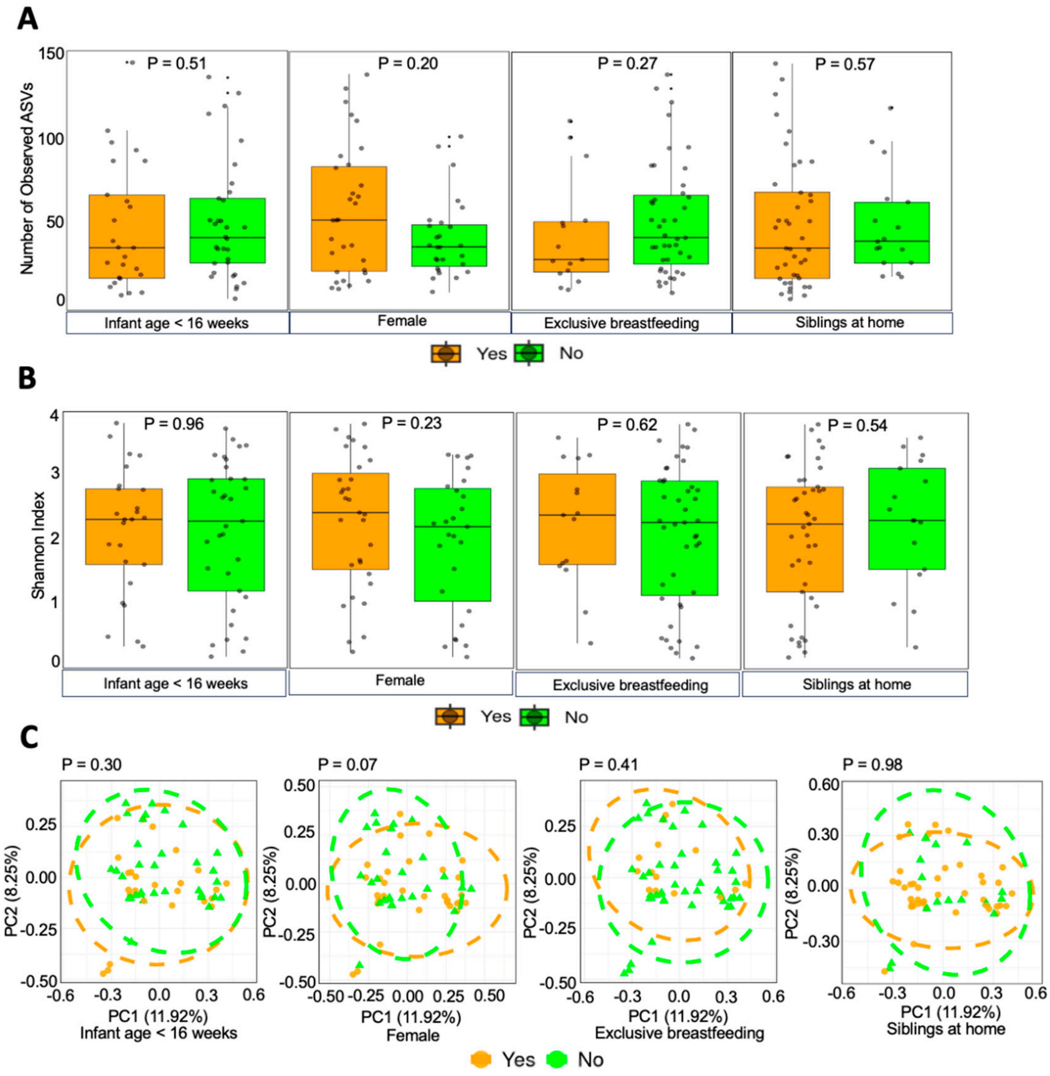

Figure S1. Evaluation of the infant nasal mycobiome by phenotype variables across the entire cohort (Year 1 group and Year 2 group) to assess potential confounding factors. **A**, Alpha-diversity analyses (observed ASV numbers) by infant age, sex, breastfeeding type, and sibling presence at home. Statistical significance was assessed using the Wilcoxon rank-sum test. **B**, Alpha-diversity analyses (Shannon index) by infant age, sex, breastfeeding type, and sibling presence at home. Statistical significance was assessed using the Wilcoxon rank-sum test. **C**, PCoA plots based on the Bray-Curtis distance, stratified by infant age, sex, breastfeeding type, and sibling presence at home. Statistical significance was analyzed with the ANOSIM test.
